# Transgenerational exposure to ocean acidification impacts the hepatic transcriptome of European sea bass (*Dicentrarchus labrax*)

**DOI:** 10.1186/s12864-023-09353-x

**Published:** 2023-06-15

**Authors:** Pauline Auffret, Arianna Servili, Anne-Alicia Gonzalez, Marie-Lou Fleury, Felix Christopher Mark, David Mazurais

**Affiliations:** 1 IFREMER, SEBIMER, Plouzané, 29280 France; 2 IFREMER, PHYTNESS, Univ Brest, CNRS, IRD, LEMAR, Plouzané, 29280 France; 3 MGX-Montpellier GenomiX, Univ. Montpellier, CNRS, INSERM, Montpellier, France; 4 Department of Integrative Ecophysiology, Alfred Wegener Institute Helmholtz Centre for Polar and Marine Research (AWI), 27570 Bremerhaven, Germany

## Abstract

Physiological effects of ocean acidification associated with elevated CO2 concentrations in seawater is the subject of numerous studies in teleost fish. While the short time within-generation impact of ocean acidification (OA) on acid-base exchange and energy metabolism is relatively well described, the effects associated with transgenerational exposure to OA are much less known. Yet, the impacts of OA can vary in time with the potential for acclimation or adaptation of a species. Previous studies in our lab demonstrated that transgenerational exposure to OA had extensive effects on the transcriptome of the olfactory epithelium of European sea bass (*Dicentrarchus labrax*), especially on genes related to ion balance, energy metabolism, immune system, synaptic plasticity, neuron excitability and wiring. In the present study, we complete the previous work by investigating the effect of transgenerational exposure to OA on the hepatic transcriptome of European sea bass. Differential gene expression analysis was performed by RNAseq technology on RNA extracted from the liver of two groups of 18 months F2 juveniles that had been exposed since spawning to the same AO conditions as their parents (F1) to either actual pH or end-of-century predicted pH levels (IPCC RCP8.5), respectively. Here we show that transgenerational exposure to OA significantly impacts the expression of 236 hepatic transcripts including genes mainly involved in inflammatory/immune responses but also in carbohydrate metabolism and cellular homeostasis. Even if this transcriptomic impact is relatively limited compared to what was shown in the olfactory system, this work confirmed that fish transgenerationally exposed to OA exhibit molecular regulation of processes related to metabolism and inflammation. Also, our data expand the up-regulation of a key gene involved in different physiological pathways including calcium homeostasis (i.e. *pthr1*), which we already observed in the olfactory epithelium, to the liver. Even if our experimental design does not allow to discriminate direct within F2 generation effects from transgenerational plasticity, these results offer the perspective of more functional analyses to determine the potential physiological impact of OA exposure on fish physiology with ecological relevance.

**Supplementary Information:**

The online version contains supplementary material available at 10.1186/s12864-023-09353-x.

## Introduction

Most fish are effective acid-base regulators so that when exposed to ocean acidification (OA), they can regulate extracellular pH. However, pH homeostasis induces elevated P_CO2_ and HCO3- (hypercapnia) in their extracellular fluids that may have physiological effects [1]. The most documented effects are probably those related to the alteration of GABAergic system, due to an inversion of the HCO3- channels across neuronal membranes, and related consequences on sensory system and behaviour [2, 3]. The large amount of energy allocated for the ion regulation process to maintain the acid-base balance in the reduced-pH environment is not associated with a clear modification of the standard and maximal metabolic rates in most of the fish species studied [4]. However, several studies indicated OA-induced regulation in energetic requirement on isolated tissues suggesting trade-off in aerobic and anaerobic metabolism within an individual [1, 5]. For example, hypercapnia caused a shift from aerobic to anaerobic pathways of carbohydrate oxidation in the muscular tissue of **G**ilthead seabream (*Sparus aurata*) associated with the tampering of hepatic oxygen consumption and key mitochondrial enzyme activities in Atlantic Cod (*Gadus morhua*) and Marbled rockcod (*Notothenia rossii*) [5–8]. The regulation of energy metabolism is also reflected in the regulation of plasma or tissue metabolite levels, as well as in the regulation of the activity and/or the gene expression of key enzymes of aerobic and anaerobic metabolic pathways. Araújo and collaborators (2018) showed that hypercapnia-induced metabolic reprogramming in the muscle of *Sparus aurata* involved the regulation of the expression of glycolysis related enzymes such as triosephosphate isomerase B (*tpisb*), glyceraldehyde-3-phosphate dehydrogenase (*g3p*) and glycogen debranching enzyme (*gde*) as well as enzymes involved in lipid metabolism (*apo1*). Such metabolic regulation can differ among tissues depending on their function according to OA-induced energy reallocation from fitness-related traits (e.g. growth, reproduction, immune defence) [9, 10]. Moreover, OA-induced metabolic effects can vary depending on the duration of OA exposure and on the capacity of fish for OA acclimation [11]. Thus, OA induced adverse effects on metabolic rates in juvenile anemonefish, *Amphiprion melanopu*, but these effects did not occur when their parents experienced the simimar OA, revealing transgenerational acclimation [12].

It has been demonstrated that the environment in which teleost exist may generate long-term changes on its immune system [13]. While studies on shellfish demonstrated that hypercapnia has a significant negative impact on the immune system (e.g. hemocyte reactive oxygen species production, hemocyte count, lysosomal content), the scarce information available for fish indicated several OA effects on physiological parameters including immune function which, may be beneficial for the tolerance of fish to pathogens [14, 15]. For example, OA stimulated the innate immunity by increasing activity of lysozyme and complement system in Atlantic halibut (*Hippoglossus hippoglossus*) [16]. Proteomic analysis in *Sparus aurata* indicated that hypercapnia induced a regulation in the expression of factors related to cell adhesion molecule production that is relevant for immune system [7]. More recently, transcriptomic effects on the olfactory epithelium of European seabass (*Dicentrarchus labrax*), including stimulation of genes involved in innate antiviral immunity (pathogen recognition receptors and interferon-stimulated genes) were observed in F2 fish transgenerationally exposed to OA [17]. These regulations were associated with a better resistance in fish challenged with betanodavirus. The modulation of the expression of genes involved in immune response was associated with the extensive regulation of genes involved in odour transduction, ionic homeostasis and in energy metabolism. This long-term OA-induced complex regulation among biological processes within the olfactory epithelium of European sea bass is likely a part of a broader adaptive physiological response that would require further attention in other tissues.

Data regarding the effects of acidification on the hepatic biological processes of fish are very scarce. Since the liver is the primary organ for metabolism, is involved in immune regulation [18] and is sensitive to a great variety of environmental factors [19, 20], we hypothesized that hepatic transcriptome could also be regulated by transgenerational exposure to OA which could have physiological consequences for fish. While the liver has an extraordinary capacity to regenerate upon various acute injuries, chronic stress such as long-term OA exposure may induce hepatic tissue disorders such as fibrosis that may interfere with normal tissue function [21, 22]. In the present study, we characterized the effects of OA on biological processes by targeting differentially expressed genes with a special focus on genes related to immune responses, metabolism and cellular homeostasis that have been revealed as regulated at transcriptomic level in metazoans [23]. This study was carried out on the same batch of animals that were subjected to transcriptomic analysis in the olfactory rosette, allowing comparison of the global transcriptomic response induced by OA between the two tissues [17].

## Materials and methods

### Animal husbandry and experimental setup

Experiments were conducted at the French Research Institute for Exploitation of the Sea (IFREMER) in Plouzané, within the facilities of the PHYTNESS Laboratory (Agreement number: B29-212-05) following the European Commission recommendation 2007/526/EC and Directive 2010/63/EU for the accommodation and care of animals used for experimental and other scientific purposes. The experiment of OA conditioning was the subject of a specific authorization issued by a French Ethics Committee for animal testing [CEEA – 074: Comité d’éthique finistérien en expérimentation animale (CEFEA): Authorization APAFIS #2,018,032,209,421,223].

**Fig. 1:**
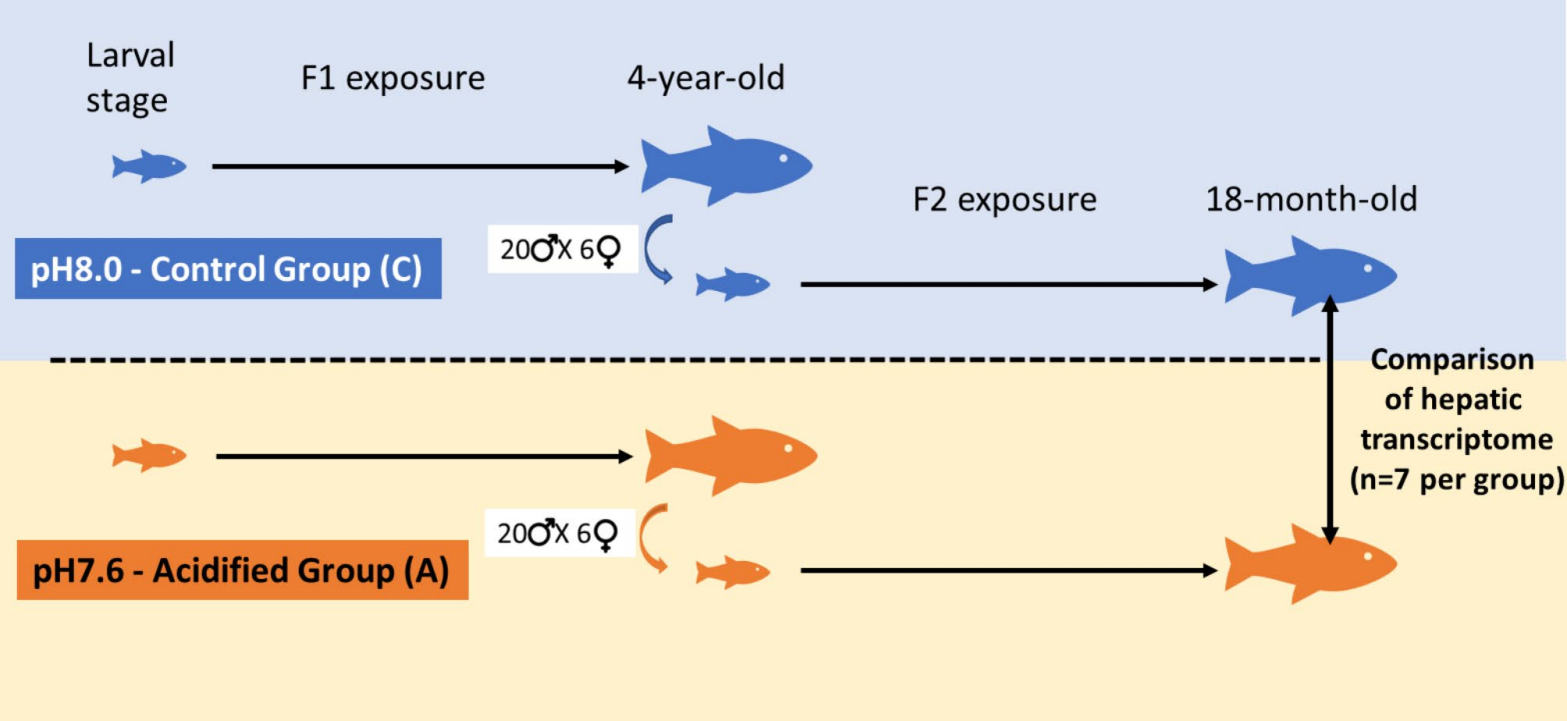
Experimental design summary. Rearing times applied on European sea bass parental linage (F1) and their offspring (F2) exposed to either the control (pH8.0, blue) or the acidified (pH7.6, orange) treatment

Animals used in this study were previously described [17]. Livers were sampled from 18 months-old F2 juveniles of European sea bass exposed from larval stage to control conditions (~ pH8.0) or to OA conditions (~ pH7.6) (Fig. 1). F2 fish were obtained from an *in vitro* fertilisation of 4 years old F1 parents (20 males X 6 females) exposed from larval to adult stages to control conditions (~ pH8.0) or to OA conditions (~ pH7.6). F2 juveniles were reared in water at the same pH as the brood stock F1 they originated from. No gene expression was performed on the immature gonads of the F2 fish and the absence of visual dimorphism did not allow us to sex them. Rearing conditions during larval and juvenile stages were similar to those described in [24]. 201 fish per treatment were distributed in triplicates in culture tanks (400 L, six tanks = three replicas per treatment) that were part of an open-circuit system. To guarantee high quality, seawater pumped 500 m off the coastline at a depth of 20 m passed through a sand filter, a tungsten heater, a degassing column packed with plastic rings, a 2-µm filter membrane, and a UV lamp. Seawater for the control treatment was then poured into each of the three replicas tanks. For each triplicate per treatment, temperature and salinity followed seasonality of the Bay of Brest. For the OA condition, CO2 was injected at constant flow in a header tank equipped with a degassing CO2 column to favour mixing and adjusted by a flow-control unit. From there, seawater flow poured hydrostatically into each of the 3 replicate tanks. pH and temperature were measured daily with a pH meter (WTW 3110; Xylem Analytics Germany, Weilheim, Germany, NBS scale). Total alkalinity was measured once a week following the adapted protocol of [25]. Summary data containing physico-chemical parameters of the rearing seawater is available in the SEANOE repository 10.17882/87395.

Fish from both conditions were fed ad libitum and no significant difference was observed in the mean body weights between the two groups [17].

### RNA extraction

Fish sampling was spread over two days during the same time period (9:00–11:00 a.m.) to avoid circadian variations. Fish from both groups were sampled each day to avoid the overlapping of group and day factors. Fish were fasted for 24 h before sampling. Fish were anesthetized (20 mg L-1), and then euthanized with a lethal dose (200 mg L-1) of tricaine methane sulfonate 222 (MS222, Pharmaq, Fordingbridge, Hampshire, UK). Full livers were collected from 15 individuals per group (5 fish per triplicate), weighed then flash frozen in liquid nitrogen and preserved at -80 °C. Total RNA was extracted from 7 individual powdered livers (ground under liquid nitrogen) per group (2 or 3 fish per triplicate). RNA extraction was performed using Extract-All reagent (Eurobio, Courtaboeuf, Essonne, France) combined with Nucleospin RNA column according to the manufacturer’s instructions (Macherey–Nagel, Düren, Germany). RNA concentration and purity were verified (260/280 ratio > 2) using an ND-1000 NanoDrop® spectrophotometer (Thermo Scientific Inc., Waltham, MA, USA) and the integrity of RNA was verified using an Agilent Bioanalyzer 2100 (Agilent Technologies Inc., Santa Clara, CA, USA). All samples showed an RNA integrity (RIN) score > 8. RNA samples were stored at − 80 °C for further RNA sequencing.

### RNA-Sequencing analysis

The 14 total RNA samples extracted from liver (7 per group) were sent to the sequencing platform GenomiX (MGX, Montpellier, France) for transcriptome analysis through RNA sequencing (RNA-Seq). RNA-seq libraries were prepared using the Truseq stranded mRNA sample prep kit (Illumina, San Diego, CA, USA) according to the manufacturer’s instructions. Briefly, polyadenylated RNAs were selected from 1 µg of total RNA using oligo-dT magnetic beads. The polyA + RNAs were fragmented using divalent cations at elevated temperature and reverse transcribed using random hexamers, Super Script II (Thermo Fisher Scientific, Carlsbad, CA) and actinomycin D. Deoxy-TTP was replaced by dUTP during the second strand synthesis to prevent its amplification by PCR. Double stranded cDNAs were adenylated at their 3’ ends and ligated to Illumina’s adapters containing unique dual indexes (UDI). Ligated cDNAs were PCR amplified for 15 cycles and the PCR products were purified using AMPure XP Beads (Beckman Coulter Genomics, Brea, CA, USA). The size distribution of the resulting libraries was monitored using a Fragment Analyzer (Agilent Technologies, Santa Clara, CA, USA) and the libraries were quantified using the KAPA Library quantification kit (Roche, Basel, Switzerland).

The libraries were denatured with NaOH, neutralized with Tris-HCl, and diluted to 150 pM. Clustering and sequencing were performed on a NovaSeq 6000 (Illumina, San Diego, CA, USA) using the single read 100 nt protocol on 2 lanes of a flow cell SP. Image analyses and base calling were performed using the NovaSeq Control Software and the Real-Time Analysis component (Illumina). Demultiplexing was performed using Illumina’s conversion software (bcl2fastq 2.202.20.0.422). The quality of the raw data was assessed using FastQC (v 0.11.9) from the Babraham Institute, multiQC v1.9 [26] and the Illumina software SAV (Sequencing Analysis Viewer). FastqScreen (v 0.15.1) was used to identify potential contamination.

### Transcriptomic and Gene Ontology (GO) analysis

Raw reads were processed using Trim Galore v0.6.7 [27] to perform quality trimming (Q = 15) and to remove the T overhang at 5’ end of reads, according to Illumina recommendations for stranded mRNA Prep protocols [28].

Trimmed reads were mapped to the European sea bass genome v1 [29] using STAR v2.7.9a [30] with standard parameters. STAR provided matrices of raw counts which were used to perform differential analysis.

To minimize the false-positive rate, the count matrices were filtered for low expressed transcripts (minimum counts > 10 in at least half of the samples). Differentially expressed genes (DEGs) between acidified and control samples were identified using DESeq2 v2.1.34 [31] with R v4.1.2 (https://www.R-project.org/) and a collection of graphics and statistics packages : rafalib [32] ; pheatmap [33]; Sartools [34] ; gplots [35] and ggplot2 [36]. DESeq2 method internally corrects for library size and uses negative binomial generalized linear models to test for differential expression. DEGs were annotated using available annotations published with GCA_000689215.1 genome release (Tine et al., 2014).

### Statistical analysis

#### Liver mass and HSI

Shapiro-Wilk normality test were performed to ensure the normality of the data related to liver mass and hepatosomatic index. Data were log transformed to allow analysis of variance (ANOVA). Fisher’s test was used to test if the variances were equal. Two-way ANOVA was performed to determine the potential effect of pH condition and sampling day on the liver mass and the hepatosomatic index, with significant p-value fixed at 0.05.

*RNAseq*: In this study the statistical design considered two factors: pH condition and sampling day. The statistical model was built using ‘counts ~ sampling_day + group’ design formula, where the qualitative variable ‘group’ indicates sample group (acidified/control). Fold changes were adjusted using Bayesian shrinkage estimators for effect sizes with apeglm package [37]. All features with adjusted p-value smaller than 0.01 (Benjamini-Hochberg method) were reported as differentially expressed (DEGs). Over- and under-representation of Gene Ontology (GO) terms associated with DEGs were tested using goatools v0.8.12 [38]. Goatools process is based on Fisher’s exact test and GO database from geneontology (go-basic.obo release 2017-07-07, https://geneontology.github.io/docs/download-ontology/).

## Results

The two way ANOVAs did not reveal any significant effect of pH condition and sampling day on the mean liver mass and hepatosomatic index of the 18-months-old juveniles (Table 1).

Among the 16.816 genes analysed, we detected 236 genes (1.4% of total) as differentially expressed in the liver of acidified (A) vs. control (C) fish groups with *p*-value ≤ 0.01, 111 and 125 genes exhibiting higher expression levels in the C and A groups, respectively (additional Tables 1 and 2). GO enrichment analysis did not reveal either biological process, molecular pathway or cell component over-represented among the differentially expressed genes. A set of 13 genes related to carbohydrate metabolism was differentially expressed between the A and C groups, 4 genes (*akr1a1*, *enosf1*, *cpt1a* and *fggy*) being up-regulated, whereas 9 genes exhibited down regulation (*pfkfb4*, *irs2*, *ppp1cc*, *g6pc2*, *ppp1r3c*, *mpi*, *adpgk*, *uxs1* and *pck1*) in the A group (Table 2).

We found 15 differentially expressed genes related to cellular homeostasis. Among the down-regulated genes in the A group we found *slc12a9*, *sox4* and *irs2* while *pth1r*, *txnrd1* while *sodc* were found up-regulated (Table 3). 46 of the 236 differentially expressed genes are involved in a biological process related to immune system process and/or inflammation (innate immune response, regulation of immune system process, inflammatory response), 17 and 29 genes being up-regulated in the A and C groups, respectively (Table 4). Several *nlcr3-like* genes exhibited significantly differential expression, 1 and 5 being up and down-regulated in the A group, respectively.

8 and 11 genes involved in cell adhesion were down- and up-regulated in group A, respectively (Table 5). Among them, fibulin-2 gene was highly down-regulated (x0.11), while plexin C-1 was highly up-regulated (x14).

Among the 236 genes differentially expressed, 90 (38% of the differentially expressed genes) were also previously found regulated in the olfactory epithelium of fish from same experimental group [17] and 58 were regulated in the same way. Among them, *pvrl1* and *pth1r* genes exhibited acidification-induced higher expression in both tissues, while the opposite was observed for *irs2*, *ppp1r3c* and *ppp1c* genes (additional Table 3).

Only 20 genes involved in various biological processes were shown differentially expressed between the two sampling days (additional Table 4). No interaction between sampling day and pH condition factors was found for any gene.

**Table 1:** ANOVA testing for differences in Liver mass Hepatosomatic Index (HSI), in response to pH condition and day of sampling (fixed factors) and interaction effects. Means ± SD in brackets

	Control group	Acidified group	pH condition (p-value)	Sampling day (p-value)	Sampling day x pH condition (p-value)
Liver mass (g) (Log 10)					
Day 1	0.42 (± 0.14)	0.38 (± 0.09)	0.66	0.99	0.77
Day 2	0.25 (± 0.33)	0.23 (± 0.21)			
HSI (Log 10)					
Day 1	-1.80 (± 0.11)	-1.84 (± 0.10)	0.42	0.34	0.93
Day 2	-1.76 (± 0.10)	-1.80 (± 0.11)			

**Table 2:** Genes involved in carbohydrate metabolism regulated (p-value ≤ 0.01) by OA in the liver of *D. labrax*

Carbohydrate metabolism	Gene symbol	seqDescription	log2Fold Change	padj
Up-regulated in A group				
	AKR1A1	alcohol dehydrogenase	0,34	7,88E-03
	ENOSF1	mitochondrial enolase superfamily member 1	0,51	2,32E-03
	CPT1A	carnitine o-palmitoyltransferase liver isoform-like isoform 1	0,57	5,36E-03
	FGGY	fggy carbohydrate kinase domain-containing	1,69	1,06E-03
Down-regulated in A group				
	PFKFB4	6-phosphofructo-2-kinase fructose- -biphosphatase 4	-1,81	4,55E-03
	IRS2	insulin receptor substrate 2-like	-1,30	7,74E-04
	PPP1CC	serine threonine-protein phosphatase pp1-gamma catalytic subunit	-1,11	4,68E-03
	G6PC2	glucose-6-phosphatase-like isoform 1	-0,96	3,02E-03
	PPP1R3C	protein phosphatase 1 regulatory subunit 3c	-0,90	3,86E-03
	MPI	mannose-6-phosphate isomerase	-0,41	1,75E-03
	ADPGK	adp-dependent glucokinase-like	-0,35	2,17E-03
	UXS1	udp-glucuronic acid decarboxylase 1-like	-0,35	7,36E-03
	PCK1	phosphoenolpyruvate cytosolic	-0,03	9,61E-03

**Table 3:** Genes involved in the process of cellular homeostasis regulated (p-value ≤ 0.01) by OA in the liver of *D. labrax*

Cellular homeostais	Gene symbol	seqDescription	log2Fold Change	padj
Up-regulated in A group				
	LDHD	probable d-lactate mitochondrial	0,61	2,01E-03
	SODC	superoxide dismutase	0,62	1,64E-04
	TXNRD1	thioredoxin reductase cytoplasmic	0,78	2,13E-03
	CROCC	rootletin isoform x1	1,28	2,13E-03
	PTH1R	parathyroid hormone parathyroid hormone-related peptide receptor-like	1,69	2,11E-03
	FGGY	fggy carbohydrate kinase domain-containing	1,69	1,06E-03
Down-regulated in A group				
	SLC12A9	solute carrier family 12 member 9-like	-1,43	8,64E-06
	SOX4	sry (sex determining region y)-box 4	-1,31	3,73E-04
	IRS2	insulin receptor substrate 2-like	-1,30	7,74E-04
	ABCB8	atp-binding cassette sub-family b member mitochondrial-like	-1,27	6,23E-08
	TMEM64	transmembrane protein 64	-1,06	1,22E-03
	CS012	protein c19orf12 homolog	-0,76	7,20E-03
	PLAA	phospholipase a-2-activating protein	-0,59	2,82E-03
	CX30.3	gap junction beta-6	-0,49	9,87E-03
	PCK1	phosphoenolpyruvate cytosolic	-0,03	9,61E-03

**Table 4:** Genes involved in the immune system and inflammation related processes regulated (p-value ≤ 0.01) by OA in the liver of *D. labrax*

Immune system and inflammation related processes	Gene symbol	seqDescription	log2Fold Change	padj
Up-regulated in A group				
positive regulation of immune system process	PBRM1	protein polybromo-1-like	0,61	1,23E-04
positive regulation of immune system process	NCK2	cytoplasmic protein nck2	0,40	7,20E-03
positive regulation of immune system process	CFHR4	complement factor h-like	1,66	6,36E-03
immune system process	NLRC3l	protein nlrc3-like	1,73	1,36E-03
immune system process	IDO2	indoleamine -dioxygenase 2	0,70	3,97E-03
immune system process	PVRL1	poliovirus receptor-related protein 4-like	0,69	7,73E-03
immune system process	DACT2	dapper homolog 2	0,50	4,99E-03
innate immune response	CD58	cd48 antigen	0,90	2,82E-09
innate immune response	SMPD1	sphingomyelin phosphodiesterase	0,46	2,32E-03
regulation of innate immune response	SLC15A2	solute carrier family 15 member 2-like	0,84	5,09E-03
regulation of innate immune response	DAK	bifunctional atp-dependent dihydroxyacetone kinase fad-amp lyase	0,79	3,62E-08
regulation of innate immune response	TNR14	tumor necrosis factor receptor superfamily member 14-like	0,59	1,81E-04
immune system process / inflammatory response	AIMP1	aminoacyl trna synthase complex-interacting multifunctional protein 1-like	1,68	2,80E-05
immune system process / negative regulation of inflammatory response	GBA	glucosylceramidase isoform x3	0,32	8,34E-02
positive regulation of immune system process / inflammatory response	SELP	selectin p (granule membrane protein antigen cd62)	1,59	5,65E-04
regulation of immune system process / negative regulation of inflammatory response	SODC	superoxide dismutase	0,62	1,64E-04
inflammatory response	NFKBIAA	nf-kappa-b inhibitor alpha	0,43	3,86E-03
Down-regulated in A group				
immune system process	NLRC3l	protein nlrc3-like	-1,68	5,83E-04
immune system process	NLRC3l	protein nlrc3-like	-1,63	2,13E-03
immune system process	RGS1	regulator of g-protein signaling 1	-1,43	3,49E-03
immune system process	NLRC3l	protein nlrc3-like	-1,25	2,13E-03
immune system process	NLRC3l	protein nlrc3-like	-1,18	9,76E-03
immune system process	SKIL	ski-like protein	-0,79	1,15E-03
immune system process	RAB35	ras-related protein rab-35-like	-0,72	5,83E-04
immune system process	NLRC3l	protein nlrc3-like	-0,71	2,56E-03
immune system process	SGPL1	sphingosine-1-phosphate lyase 1	-0,50	1,51E-03
immune system process	ROMO1	reactive oxygen species modulator 1-like	-0,24	7,47E-03
inflammatory response	PLAA	phospholipase a-2-activating protein	-0,59	2,82E-03
immune system process / inflammatory response	HP	haptoglobin isoform x1	-0,41	2,17E-03
innate immune response	MPI	mannose-6-phosphate isomerase	-0,41	1,75E-03
regulation of innate immune response / negative regulation of inflammatory response	SRC	proto-oncogene tyrosine-protein kinase src-like	-1,61	1,77E-08
positive regulation of immune system process	SOX4	sry (sex determining region y)-box 4	-1,31	3,73E-04
positive regulation of immune system process	IRS2	insulin receptor substrate 2-like	-1,30	7,74E-04
positive regulation of immune system process	TMEM64	transmembrane protein 64	-1,06	1,22E-03
positive regulation of immune system process	ACTB	cytoplasmic 1-like	-0,78	9,31E-03
positive regulation of immune system process	TISB	butyrate response factor 1	-0,76	9,22E-04
positive regulation of immune system process	ACTL6A	actin-like protein 6a-like isoform 1	-0,60	1,51E-03
positive regulation of immune system process	PCK1	phosphoenolpyruvate cytosolic	-0,03	9,61E-03
positive regulation of immune system process / positive regulation of inflammatory response	PLA2G7	platelet-activating factor acetylhydrolase	-0,75	3,19E-05
positive regulation of immune system process / positive regulation of inflammatory response	SERPINE1	plasminogen activator inhibitor 1 precursor	-0,10	7,36E-03
regulation of immune response / regulation of inflammatory response	ER	estrogen receptor alpha	-0,24	9,76E-03
positive regulation of immune system process / regulation of inflammatory response	IL1R1	interleukin-1 receptor type 1 precursor	-1,45	2,72E-04
positive regulation of innate immune response	MFAP4	microfibril-associated glycoprotein 4-like	-1,99	5,65E-06
negative regulation of innate immune response	ZDHHC18	palmitoyltransferase zdhhc18-like	-0,39	3,77E-03
negative regulation of innate immune response	PARP14	poly(ADP-Ribose) Polymerase Family Member 14	-1,52	3,32E-03
inflammatory response	ATRN	attractin	-0,22	6,36E-03

**Table 5:** Genes involved in the process of cell adhesion regulated (p-value ≤ 0.01) by OA in the liver of *D. labrax*

Cell adhesion	Gene symbol	seqDescription	log2Fold Change	padj
Up-regulated in A group				
	NCK2	cytoplasmic protein nck2	0,40	7,20E-03
	DACT2	dapper homolog 2	0,50	4,99E-03
	CLASP1	clip-associating protein 1 isoform x1	0,51	4,50E-04
	TNR14	tumor necrosis factor receptor superfamily member 14-like	0,59	1,81E-04
	PBRM1	protein polybromo-1-like	0,61	1,23E-04
	PLXNA4	plexin-a4 isoform x2	0,69	8,60E-03
	LAMA3	laminin subunit alpha-3	0,72	1,22E-03
	FZD4	frizzled-4-like	0,98	3,03E-03
	CORO1C	coronin-1c isoform 2	1,10	4,50E-04
	SELP	selectin p (granule membrane protein antigen cd62)	1,59	5,65E-04
	PLXNC1	plexin-c1	3,88	2,13E-03
Down-regulated in A group				
	FBLN2	fibulin-2 isoform 1	-3,13	2,82E-09
	SRC	proto-oncogene tyrosine-protein kinase src-like	-1,61	1,77E-08
	SOX4	sry (sex determining region y)-box 4	-1,31	3,73E-04
	ANK3	ankyrin-3-like isoform x1	-1,21	3,30E-10
	ACTB	cytoplasmic 1-like	-0,78	9,31E-03
	ACTL6A	actin-like protein 6a-like isoform 1	-0,60	1,51E-03
	SERPINE1	plasminogen activator inhibitor 1 precursor	-0,10	7,36E-03
	PCK1	phosphoenolpyruvate cytosolic	-0,03	9,61E-03

## Discussion

This study reports the effects of transgenerational OA exposure on the hepatic transcriptome of European sea bass. We assume that the experimental protocol used in the present study did not allow to investigate the transgenerational plasticity to OA since hepatic transcriptome was not parallelly analysed in F2 juveniles whose parents had not been previously exposed to OA. Some studies revealed positive transgenerational acclimation of fish to OA since OA-induced decrease in the growth rate or survival of juvenile was compensated when parents also experienced similar conditions [12, 39]. In contrary, negative transgenerational effects of OA exposure were observed in terms of survival and body size in marine three-spined stickleback (*Gasterosteus aculeatus*) when parents were acclimated to the high-CO2 environment [40]. Even if our results do not allow to distinguish direct effect of OA on F2 hepatic transcriptome from transgenerational effects of parents’ previous exposure, they reveal the impact of OA on hepatic transcriptome considering the transgenerational exposure of fish to a realistic scenario of future ocean acidification, which is of ecological relevance.

In order to limit false positive differentially expressed genes, only RNAs with adjusted *p-value* below 0.01 were retained as differentially expressed, which ensures robustness of our data. Only 20 genes involved in a wide range of biological processes were shown differentially expressed between the sampling days. No relevant scientific information can be derived from the regulation of so few genes and these data will not be discussed.

Our results displayed 236 genes significantly differentially expressed by OA. This number of differentially expressed genes is relatively low compared to the 9112 found differentially expressed with the same adjusted p-value ≤ 0.01 in the olfactory rosette of the same fish batch under the same conditions [17]. This difference can be explained, at least in part, by the fact that the olfactory rosette is in direct contact with the surrounding environment, therefore it continuously activates mechanisms for regulating ion exchange that interfere with other biological processes related to metabolism and neural activity. Accordingly, we only found one gene with potential function in inorganic ion homeostasis (*slc12a9*) regulated by OA in the liver [41]. This data confirms the efficiency of fish to compensate for a hypercapnic acid-base disturbance through regulation of ion exchange occurring chiefly in water exposed epithelia [1, 42]. In the same way, while we cannot rule out post transcriptional regulation of genes and associated differential activity of metabolic pathways in the liver, our data indicated limited transcriptomic effects of transgenerational exposure to OA on the hepatic metabolic pathways in European sea bass. Since the liver is a metabolic overachiever organ which governs energy balances in the body and that tissues were sampled in unstimulated and fasted individuals, this data suggests limited long-term effects of OA on the global energy metabolism of resting European sea bass. This result agrees with a previous study indicating that 1.5 years exposure to OA did not affect fish standard metabolic rate (SMR) in European sea bass [43]. Moreover, in this latter study, authors revealed that OA induced higher aerobic capacities (maximal metabolite rates) and concluded by asking about the potential impact of OA on anaerobic metabolic capacity. Anaerobic capacity of fish depends, among other, on their ability to use glucose as substrate through anaerobic glycolysis. Previous studies performed in gilthead seabream revealed that the intermediary metabolism, especially the balance between aerobic and anaerobic metabolism pathways, was reorganized in the liver of fish exposed five weeks to hypercapnia in order to maintain systemic ion homeostasis (Ruiz-Jarabo et al., 2021). In the present study, 13 genes (5% of the differentially expressed genes) involved in carbohydrate metabolism were found differentially expressed. Among them, some genes promoting glycolysis (*pfkfb4*, *irs2* and *adpgk*) and regulating glycogen metabolism (*ppp1cc*, *ppp1r3*) were found down-regulated, suggesting an OA-induced long term discrete fine-tuning regulation of glucose homeostasis in the liver [44–47]. According to that, we found the gene coding for the PTHrP receptor (*pth1r*) significantly up-regulated in the liver of fish exposed to hypercapnia. Interestingly, PTHrP has a role in the regulation of hepatic gluconeogenesis in European sea bass where it is supposed to promote hepatic glucose export to peripheral tissues [48]. It is noteworthy that part of the genes (e.g. *pth1r, ppp1cc*, *ppp1r3*) related to carbohydrate metabolism we found regulated in the liver was similarly regulated in the olfactory rosette, suggesting comparable OA-induced regulation of cellular pathways among different tissues of fish. In line with the potential regulation of glucose export from the liver, we observed an up-regulation (p value < 0.05, additional Table 1) of the mRNA coding for glucose transporter 2 (*glut2*) in the fish exposed to OA [49]. The liver is a relevant tissue for glycogen storage and has a major gluconeogenic function regulating blood glucose levels. Therefore the up and down-regulation of carbohydrate metabolic pathway genes we observed may be associated with regulation of glycemia and anaerobic capacity. Since the hepatic glycogen and plasmatic glucose levels were not analysed in the present study, we are not able to determine whether the transcriptomic regulation was associated with a perturbation of the blood glucose content. Previous study in European sea bass indicated that while higher circulating glucose levels were observed after short term exposure to hypercapnia, no difference was observed after several weeks of acclimation [50]. Potential regulation of anaerobic capacity may be revealed through analysis of fish performance in environment with O2 concentration below the critical oxygen level (the oxygen threshold below which SMR is no longer sustainable aerobically).

The regulation of *pth1r* gene that is essential for calcium mobilization, vitellogenesis or cortisol production in other tissues [51] may have consequence on fish physiology including bone mineralization, sexual maturation or stress response. According to that, Di Santo (2019) found changes in mineralization in the skeleton of *L. erinacea* [52]. Moreover, a recent study performed in European sea bass indicates that OA alters the physiological response to acute stress via the neuroendocrine regulation of the corticotropic axis [53]. Also, recent data revealed that OA regulates the reproductive function both in terms of sexual maturation and gamete quality [54]. Interestingly, we observed in the present study a down-regulation in the liver of fish exposed to OA of the gene coding estradiol receptor alpha that are responsible for the liver production of vitellogenins. We are fully aware that the regulation of the *pth1r* gene cannot be formally associated with the regulation of these physiological functions in other tissues of fish long-term exposed to OA, but our result offer possibilities for further investigation that also call for a comparison of metabolomes.

The very few genes (i.e. *cpt1a*) involved in lipid metabolism exhibiting differential expression in the present study confirmed previous data obtained in cod showing the resilience of lipid metabolism for fish when exposed for long time to elevated pCO2 [55].

Our data revealed also several differentially expressed genes involved in cellular homeostasis suggesting regulation in cellular mechanisms for maintaining normal liver function in fish transgenerationally exposed to OA. Especially, two genes involved in redox homeostasis (*txnrd1* and *sodc)* were found up-regulated in the liver of fish exposed to OA which is consistent with the associated increase in *akr1a1* gene in yellow catfish, whose expressions has been associated with the regulation of oxidative status [56]. While the present data suggest that transgenerational exposure to OA may be associated to the regulation of molecular mechanisms involved in hepatic antioxidant defence, further analyses including measurements of antioxidant enzyme activities and intra-tissue ROS concentrations would be required to assess the actual oxidative status of the hepatic tissue. That would be of special interest since the production of ROS interacts with numerous biological processes including pathogen destruction by the innate immune system [57, 58] and the OA-induced regulation of ROS production in shellfish has been associated with a regulation of the immune system [14, 15].

Among the differentially expressed genes in the liver, 20% are directly or indirectly involved in the immune or inflammatory responses. These observations revealed an effect of OA in the fish immune mechanisms with the regulation of important pro- and anti-inflammatory mediators. The down-regulation of genes involved in pro-inflammatory response (*atrn, hp, il1r1, plaa, pla2g7, serpine1)* combined with up-regulation of genes involved in anti-inflammatory response (*gba*, *nfkbiaa*, *sodc*) suggest a fine tune regulation of the balanced pro and anti-inflammatory response. Inflammation is a characteristic feature of the hepatic wound-healing response to injury that may be beneficial in the short term for example by inducing immune responses that lead to the eradication of pathogens, but may be at long term linked to the development of fibrosis. Interestingly, our data revealed the down-regulation of *mpi* whose expression level is considered in vertebrates, including fish, inversely correlated with liver fibrosis [59, 60]. The liver fibrosis is characterized by the synthesis and altered deposition of extracellular matrix components. Again, the regulation of the *mpi* gene expression alone is not sufficient to suggest that OA regulates the fibrotic state of the liver, but together with the regulation of genes involved in cell adhesion (e.g. *fbln2*) that we observed in the present study, this data opens up the prospect of further functional studies to address the potential long-term impact of OA on biological processes related to liver fibrogenesis.

In line with the regulations observed on the expression of genes involved in the inflammatory response, we found several genes involved in the regulation of immune system process down-regulated in the liver of fish exposed to OA (*sox4, irs2, tmem64, actb, tisb, actl6a, pck1, pla2g7, serpine1, il1r1, mfap4*). Combined with the OA-induced up-regulation of other genes involved in the positive regulation of immune system such as factors involved in complement system (*cfhr4*), those results confirm previous data observed in the olfactory rosette of the same fish indicating molecular regulation by OA of the immune function [17]. While *nrlc3l* genes expression are regulated in both the liver and the rosette, we did not find many regulated hepatic genes involved in pathogen recognition receptors (except for *pvrl1*) and interferon-stimulated genes suggesting OA-induced tissue specific regulation of immune process. Interestingly, previous data obtained in other fish species revealed that OA stimulated the innate immunity by increasing activity of complement system and by regulating the expression of factors related to cell adhesion molecule [7, 16]. It would be interesting to determine whether this present expressional hepatic profile is correlated to difference in resistance to pathogens such as *V. anguillarum* which affect different organs including liver in European sea bass [61].

In conclusion, the present data revealed limited OA-induced regulation of the hepatic transcriptome following transgenerational exposure to an IPCC RCP8.5 scenario. Even limited, this plasticity is a component of molecular phenotypic acclimation of European sea bass to OA. Given the regulation occurring on the expression of genes involved in the metabolism, inflammatory and immune response observed in the present study, the question remains if these differences in phenotypes are associated with a different physiological status that may impact on the fitness of the animals. Further experiments addressing the physiological performance of the fish (sexual maturation, tolerance to pathogen, fish health status) as well as tissue-specific reaction norms towards the various climate change related drivers would greatly facilitate the ecological interpretation of this plasticity in the context of the global climate crisis.

## Electronic supplementary material

Below is the link to the electronic supplementary material.

Supplementary Material 1

Supplementary Material 2

Supplementary Material 3

Supplementary Material 4

## Data Availability

Summary data containing physico-chemical parameters of the rearing seawater is available in the SEANOE repository 10.17882/87395. Raw reads have been published to ENA, under the accession project number PRJEB59362. Analysis scripts used in this study are publicly available at https://gitlab.ifremer.fr/bioinfo/bioanalysis/public/livacid.
